# N-Doped Biochar from Lignocellulosic Biomass for Preparation of Adsorbent: Characterization, Kinetics and Application

**DOI:** 10.3390/polym14183889

**Published:** 2022-09-17

**Authors:** Jing Li, Fanxun Lv, Ran Yang, Liping Zhang, Wei Tao, Guotao Liu, Hui Gao, Ying Guan

**Affiliations:** School of Forestry and Landscape Architecture, Anhui Agricultural University, Hefei 230036, China

**Keywords:** urea modification, efficient adsorption, porous biochar

## Abstract

*Medulla tetrapanacis* is composed of a lignocellulosic biopolymer and has a regular porous structure, which makes it a potential biomass material for preparing porous N-doped biochar. Herewith, N-doped *Medulla tetrapanacis* biochar (UBC) was successfully prepared by modification with urea and NaHCO_3_ under pyrolysis at 700 °C. The nitrogen-containing groups were efficiently introduced into biochar, and the micro-pore structures of the UBC were developed with sizeable specific surface area, which was loaded with massive adsorption sites. The adsorption kinetics and isotherms of the UBC conformed to pseudo-second-order and Langmuir model. The superior adsorption capacities of the UBC for methylene blue (MB) and congo red (CR) were 923.0 mg/g and 728.0 mg/g, and the capacities for Cu^2+^ and Pb^2+^ were 468.5 mg/g and 1466.5 mg/g, respectively. Moreover, the UBC had a stronger affinity for Cr^3+^ and Fe^3+^ in multiple metal ions and retained at a preferable adsorption performance for dyes and heavy metals after five cycles. Precipitation, complexation, and physical adsorption were the main mechanisms of the UBC-adsorbing metal ions and dyes. Thus, lignocellulosic biochar has great potential for removing dyes and heavy metals in aqueous solutions.

## 1. Introduction

Lignocellulose is abundant in agroforestry biomass, which replaces fossil raw materials and effectively alleviates the excessive shortage of fossil resources due to the advantage of being environmentally degradable, nontoxic, and cheap [1,2]. Lignocellulosic materials are widely used in paper and pulp industry, food, feed, energy, and fuel [2,3]. Globally, a higher utilization value of lignocellulose has been explored, such as ethanol conversion, biological medicine, and biomass pyrolysis technology [3,4]. Among various applications, the pyrolysis of lignocellulosic biomass to produce an adsorbent has attracted attention [5]. Compared with inorganic adsorbents, the low cost, good stability, high recycling rate, rich structure, and functional groups were highlighted in lignocellulosic adsorbents, which make it easier to realize industrial production [6].

*Medulla tetrapanacis,* as a biopolymer lignocellulosic material, has a content of holocellulose of more than 80%. *Medulla tetrapanacis* is cylindrical, white in color, lightweight, central with a translucent circular diaphragm, and has a regular porous structure [7,8,9]. However, there is little research on its high value-added applications, especially the properties based on its porous structure and abundant lignocellulose. In the previous study, mesoporous biochar was prepared from *Medulla tetrapanacis*, which had fast kinetics and good regeneration properties and exhibited excellent adsorption capacity for Cu^2+^ (458.72 mg/g) and Pb^2+^ (1031.23 mg/g) [8,10]. In contrast, the research about the adsorption of dyes using the biochar from *Medulla tetrapanacis* are rarely reported. It would be interesting to further investigate the adsorption properties of this biopolymer lignocellulosic material.

Lignocellulosic biochar adsorbent has acquired growing interest due to its cost-effectiveness, acid and alkali resistance, high temperature resistance, environmental friendliness, economy, and convenient operation [11,12,13]. It is generated through the pyrolysis of lignocellulosic biopolymer resources under certain temperature (<900 °C) with anoxic conditions, and presents a loose and porous morphology with a large specific surface area and mostly amorphous structure [14,15]. Furthermore, a lot of hydroxyl, carboxyl, amino, carbonyl, and other functional groups exposed on the biochar surface provide number of adsorption sites [16,17]. Abundant pore structures, large specific surface areas, and active functional groups are important parameters that determine the adsorption properties of adsorbent. In contrast, the adsorption effectivity and adsorption capacity of unmodified biochar suffer from some limitations [12,18]. Therefore, chemical modification and composite or physical methods are usually required to improve the physicochemical properties of an adsorbent, thereby the adsorption performance for pollution is enhanced [19]. The corn straw biochar was combined with ZnO/ZnS nanoparticles to obtain a nanocomposite adsorbent with a size-dependent and sizable surface area, which greatly improved the removal of Cu^2+^, Pb^2+^, and Cr^6+^ [20].

Non-metallic atoms (nitrogen, boron, and sulfur) doping has been widely used in the field of modified biochar over the last years [21,22]. Therein, N-doped is an effective method to improve the surface area, pore structure, surface chemical properties, mechanical strength, physical and chemical stabilities, and adsorption capacity for biochar modification [23,24]. Researchers have successfully prepared various N-doped biochars from *camellia sinesis*, corn straw, sawdust, and other biomass materials, which were significantly enhanced with the removal capacity of heavy metal ions [25,26,27,28]. Urea and NaHCO_3_ are a green nitrogenous compound and green activator, respectively. The ammonia and CO_2_ would be produced when they are coordinatelu used in pyrolysis, which has the dual effects of hole expansion and nitrogen doping with biomass [13,24]. The N-doped porous bamboo was prepared by urea modification through in-situ pyrolysis, and it was found that the formed pores were promoted by a small ratio of nitrogen source and activator [29]. The above research confirmed that N-doped biochar had an affinity for organic dyes and heavy metal ions.

Herein, the urea-modified biochar (UBC) was prepared by pyrolysis at 700 °C from *Medulla tetrapanacis* with urea as a nitrogen source and NaHCO_3_ as activating agents. The adsorption properties of N-doped biochar on organic dyes and heavy metal ions under a variety of influencing factors (solution concentration, reaction time and pH value) were investigated. Based on the adsorption data, the preferable adsorption capacity and the optimal process conditions of the UBC were determined, and the adsorption mechanism was explored. The competitive adsorption for five heavy metal ions and regeneration experiment were carried out to predict the application potential of UBC in wastewater adsorption, and thus expand the diversification and high value-added utilization of lignocellulosic biochar.

## 2. Materials and Methods

### 2.1. Materials

*Medulla tetrapanacis* was produced from Sichuan Province, China. Sodium bicarbonate (NaHCO_3_), sodium hydroxide (NaOH), methylene blue (MB, AR), congo red (CR, AR), hydrochloric acid (HCl, 36~38 wt%), and urea (CO(NH_2_)_2_) were purchased from Shanghai Aladdin Co., Ltd. (Shanghai, China). Heavy metal ions were used from Shanghai Macklin Biochemical Co., Ltd. (Shanghai, China), including cadmium nitrate tetrahydrate (Cd (NO_3_)_2_·4H_2_O), lead nitrate (Pb (NO_3_)_2_), anhydrous ferric chloride (FeCl_3_), copper nitrate (Cu (NO_3_)_2_), and chromium nitrate (Cr (NO_3_)_3_). The other reagents are analytical grade.

### 2.2. Preparation of N-Doped Biochars

*Medulla tetrapanacis* was crushed and filtered to obtain 1–2 mm size of particles. The particle was mixed with NaHCO_3_ and urea in the mass ratio of 1:1:1 and 1:2:2. The mixture was pyrolyzed at 700 °C for 2 h with a high temperature vacuum furnace (BTF-1200C, BEQ, Hefei, China) under the protection of nitrogen with the heating and cooling rate of 5 °C/min. Then, the obtained pre-products were washed with hydrochloric acid solution at 60 °C for 30 min and this process was repeated three times. The urea-modified samples were obtained after drying and sieved through a 75 μm sieve. The obtained samples with N-doped biochars were named UBC_1−1_ and UBC_1−2_, respectively. The biochar prepared without N-doped biochars at 700 °C by the same method as a control sample was recorded as BC.

### 2.3. Characterization of Biochar

The element composition and binding energies were apparent from X-ray photoelectron spectroscopy (XPS, ESCALAB 250Xi, Thermo Fisher, Waltham, MA, USA). Carbon, hydrogen, nitrogen, and oxygen contents in biochar were characterized by the fully automatic elemental analyzer (Vario EL Cube, Langenselbold, Germany). The pore structure was determined with an automatic specific surface area and pore size analyzer (ASAP2460, Micromeritics, Norcross, GA, USA). The microscopic morphological structure was detected by a field emission scanning electron microscope (SEM, Hitachi S-4800, Hitachi, Tokyo, Japan) assisted with energy dispersive spectrometer spectroscopy (EDS, X-Max N 150). The chemical structure of biochar was obtained with Fourier transform infrared spectroscopy (FTIR, Bruker Tensor II, Bruker, Karlsruhe, Germany) in the wavenumber range of 400–4000 cm^−1^ with 1% KBr and the resolution of 2 cm^−1^. The concentration of MB and CR in the solution was determined by UV-vis spectrophotometer (TU-1810PC, PERSEE, Beijing, China) at 665 nm and 500 nm, respectively. The concentrations of Cu^2+^ and Pb^2+^ in the solutions were detected by atomic absorption spectrophotometer (TAS-990, PERSEE, Beijing, China).

### 2.4. Adsorption Experiments

The adsorption properties of biochar for MB (200 mg/L), CR (200 mg/L), Cu^2+^ (200 mg/L), and Pb^2+^ (300 mg/L) were studied by batch adsorption experiments. Ten milligrams of biochar and 50 mL solution of organic dyes or heavy metal ions were carried out in conical flasks and shaken in water bath with 120 rpm at 30 °C for 24 h, and each series of adsorption experiments was repeated three times. The adsorbent was filtered after adsorption, and the series of MB and CR filtrates were diluted 10 and 20 times, respectively. The absorbance of 2 mL solution was determined by UV-vis spectrophotometer and the concentration of heavy metal ions was determined by atomic absorption spectrophotometer with 20 mL solution.

The adsorption kinetics of biochar for MB, CR, Cu^2+^, and Pb^2+^ were investigated at different pH, and the optimal pH for adsorption was obtained. The pH of dyes (3.0–9.0) and metal ions (3.0–5.5) were adjusted using 0.1 mol/L NaOH and HCl. The adsorption capacity of biochar was carried out with different initial concentrations of organic dyes or heavy metal ions (50, 100, 150, 200, 300, 400, and 500 mg/L). The adsorption kinetics of UBC for MB and CR were measured at 3, 5, 10, 15, 30, 60, 120, 240, 360, 1080, and 1440 min, respectively. The adsorption kinetics for Cu^2+^ and Pb^2+^ were measured at 3, 5, 10, 20, 30, 60, 90, 120, 180, 240, and 360 min, respectively.

The removal efficiency and the capacity of equilibrium adsorption (*Q_e_*) were calculated as follows in Equations [10]:

Removal (%) = (1 − C_e_/C_0_) × 100% (1)

*Q_e_* = (C_0_ − C_e_) V/m (2)

where, C_0_ and C_e_ are the initial and adsorption equilibrium concentrations of MB, CR, Cu^2+^, and Pb^2+^, mg/L; V represents the volume of aqueous solution, mL; and m is the dose of the adsorbent, g.

The adsorption kinetics, isotherm models, and thermodynamics model of UBC adsorption are listed in Table S1 to study the adsorption process and mechanism of UBC for metal ions and dyes.

### 2.5. Competitive Adsorption of Multi-Metal

The competitive adsorption performance of UBC was investigated and it was found that 10 mg UBC_1−2_ was taken into 50 mL of Cu^2+^, Pb^2+^, Cd^2+^, Cr^3+^, and Fe^3+^ (100 mg/L) mixed solution with the above five metal ions at pH = 5.5. Then, the system was shaken after 24 h at 30 °C.

### 2.6. Regeneration Experiments

After adsorption pollutants, the UBC adsorbent was desorbed with 0.1 mol/L HCl solution. The adsorption-desorption experiments were conducted in five cycles to detect the recycling ability of UBC.

## 3. Results and Discussion

### 3.1. Characterization of Biochars

**Figure 1:**
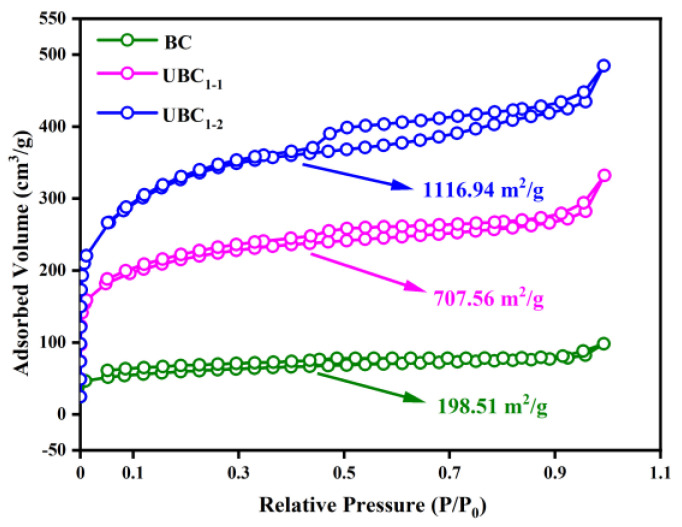
The N_2_ adsorption−desorption isotherms of BC, UBC_1−1_, and UBC_1−2_.

The morphologies of the three biochars were shown in scanning electron microscopy images (Figure S1). The BC exhibited a wrinkled morphology with less pores, and the porous microstructure was present in UBC. With the urea ratio increased, the pores of UBC_1−2_ were smaller and denser than that of UBC_1−1_. These pore characteristics contributed to the efficient dispersion and adsorption of adsorbate. The increasing porosity of UBC might be due to the cooperative effect of urea and NaHCO_3_ during pyrolysis [27,29]. The N_2_ adsorption-desorption isotherms of biochar showed that the UBC_1−1_ and UBC_1−2_ were type IV with type-H_1_ hysteresis loop indicating the mesoporous structure existed in UBC [13,30] (Figure 1). The BET surface area of biochar was evidently increased from 198.51 to 1116.94 m^2^/g during the N-doping process at the pyrolysis. The abundant pore structure and larger BET surface area of the UBC might be attributed to the urea decomposition and efficient activation of NaHCO_3_. The etching and pore expansion of biochar were effected by multiple gases produced by decomposition of urea and NaHCO_3_ [13,31]. These results with the porous construction of biochar corresponded to the SEM observations.

The N content of the UBC was significantly increased with the doping of urea, which indicated that nitrogen was successfully doped into biochar (Table 1). The electronegativity of biochar could be reduced with the introduction of nitrogen-containing functional groups and the formation of π-π bonds; the adsorption of Lewis acids and bases with organic dyes could be promoted, thereby the adsorption capacity was improved [32].

The difference of surface functional groups between the BC and UBCs were observed by FTIR spectra (Figure S2a). There was a common absorption peak at 1588 cm^−1^ of the BC and UBC, attributed to bending vibrations of −COOH [10]. A new absorption peak at 1225 cm^−1^ of UBC was presented, which was ascribed to the stretching vibration of C−N functional groups. The 786 cm^−1^ absorption peak was assigned to the bending vibrations of N−H out-of-plane [28]. The 1025–1262 cm^−1^ absorption peaks disappeared after modification by urea and originated from the bending vibrations of C−H [17,33]. These changes confirmed that N has been successfully doped into biochar, and chemical reactions occurred on the biochar surface.

### 3.2. The Influencing Factors on the Adsorption of Biochar

#### 3.2.1. The Influence of pH

**Figure 2:**
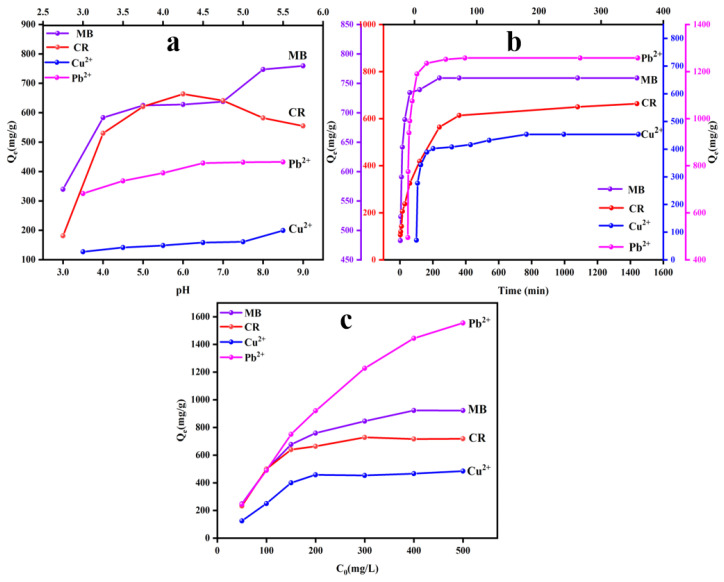
The influence factors of adsorption performances of UBC for MB, CR, Cu^2+^, and Pb^2+^: pH (**a**); adsorption time (**b**) and solution concentration (**c**).

The surface charge of the adsorbent, heavy metal ions, and dyes would be affected by the pH of the system solution [34]. As presented in Figure 2a, the adsorption capacities of UBC for MB was gradually increased with the increase of pH value (3.0–9.0). The adsorption capacity of UBC for CR was increased to its maximum value at pH = 6. When the pH value was above 6.0, the adsorption capacity of UBC exhibited a downward trend because of the negative charge of the UBC with the deprotonation, which inhibited the adsorption of CR [29,35]. As the pH increased from 2.0 to 5.5, the adsorption capacities of the Cu^2+^ and Pb^2+^ ions significantly increased. The electrostatic repulsion of protonated surface functional groups of UBC impeded the adsorption for Cu^2+^ and Pb^2+^ under strong acid condition. With the increase of the pH value, the complexation of the UBC surface with metal ions increased, and the adsorption capacity of biochar increased [36,37]. However, excessive alkalinity might lead to the generation of hydroxide anion complexes to hinder the adsorption [38]. Thus, the adsorption experiments were carried out in different acidic and alkaline environments (MB: pH = 9, CR: pH = 6, Cu^2+^ and Pb^2+^: pH = 5.5).

#### 3.2.2. The Influence of Time and Adsorption Kinetics

With the increase of adsorption time, the adsorption rate was gradually increased until it reached saturation adsorption (Figure 2b). However, the saturated adsorption time of UBC for CR was longer than MB, which might be due to the repulsive electrostatic between negative charges of CR and surface-active sites of UBC [31]. As for heavy metal ions, the saturated adsorption time for Cu^2+^ was also longer than that of Pb^2+^, resulting because the affinity of biochar for Pb^2+^ is stronger than that of Cu^2+^ [10,30].

**Figure 3:**
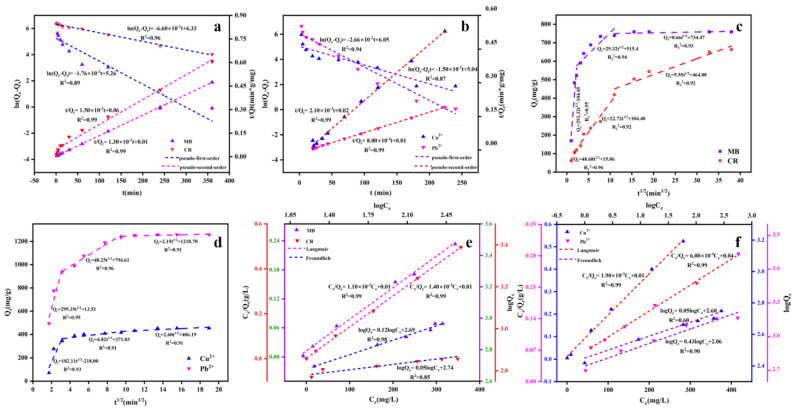
The linear plot of pseudo−first−order and pseudo−second−order adsorption kinetics by UBC: MB and CR (**a**), Cu^2+^ and Pb^2+^ (**b**). The intraparticle diffusion model: MB and CR (**c**), Cu^2+^ and Pb^2+^ (**d**); the adsorption isotherm of Langmuir and Freundlich by UBC: MB and CR (**e**), Cu^2+^ and Pb^2+^ (**f**).

As shown by the fitting coefficients in Table 2, the pseudo-second-order model was more suitable to describing the adsorption process. Therefore, the adsorption process might be controlled by chemical adsorption [39]. The fitting curves of intraparticle diffusion showed three stages of the adsorption process (Figure 3a–d). The rapid adsorption in the first stage was related to surface diffusion of organic dyes and heavy metal ions on UBC. The adsorption was slowed down in the second stage, which might be due to the diffusion process of organic dyes and heavy metal ions from the surface of biochar to the internal pores. In the third stages, the adsorption process gradually reached equilibrium due to the decrease of residual adsorption sites and the number of adsorbents [13]. The fitting curves of intraparticle diffusion model did not pass the origin, indicating there might be other adsorption processes.

#### 3.2.3. The Influence of Concentration and Adsorption Isotherms

The effect of initial concentrations of the heavy metal ions and dyes on biochar adsorption was presented in Figure 2c. With the increase of the solution concentrations, the adsorption capacities for Pb^2+^, Cu^2+^, MB, and CR of UBC gradually increased, and then reached equilibrium. As the solution concentration increased, the difference of surface concentration on both sides of the biochar was increased, which provided a higher driving force to overcome mass transfer resistance of the solution, and thus increased the adsorption capacity for heavy metal ions and dyes [13,26]. However, a limited amount of adsorbent means a limited number of adsorption sites. The limited adsorption sites of biochar would be fully occupied, and the adsorption reached equilibrium when heavy metal ions or dyes reached a certain concentration.

As shown in Figure 3e–f and Table 3, the corresponding correlation coefficient (R^2^) for MB, CR, Cu^2+^, and Pb^2+^ by UBC of the Langmuir model were higher than the Freundlich model. Hence, the adsorption isothermal was in accord with the Langmuir isotherm model, which indicated that the adsorptions for heavy metal ions and dyes by UBC were uniform monolayer adsorption [28,36].

### 3.3. Adsorption Thermodynamics

The thermodynamic parameters of the UBC adsorption process were derived from Van′t Hoff equation as shown in Table 4. The equilibrium constants K_c_ of MB and Pb^2+^ increased with the increase of adsorption temperature, indicating that the adsorption process could obtain larger adsorption equilibrium capacity at higher temperature. The above might be due to the fact that the heat provided by the temperature rise was converted into kinetic energy, which promoted the rapid movement of adsorbate particles. The K_c_ values of CR and Cu^2+^ decreased with the increase of temperature, which was related to the increase of desorption rate during adsorption [19].

The adsorption process for dyes and metal ions by UBC was spontaneous and feasible from the ∆G^θ^ value less than 0 in Table 4. The adsorption capacity of UBC for Pb^2+^ was increased with the increase of temperature, and the adsorption capacity for MB, CR, and Cu^2+^ was maintained at relatively low temperature, which might be related to the complexation and precipitation between Pb^2+^ and weak acid ions promoted by the increase of temperature on the surface of UBC [20,40]. The positive value of ∆H^θ^ demonstrated that the adsorptions of UBC for dyes and Cu^2+^ were endothermic reaction, however, the adsorption process for Pb^2+^ was exothermic. The positive value of ∆S^θ^ indicated that the disorder of adsorption environment was increased, which was attributed to the good affinity of biochar with metal ions and dyes.

### 3.4. Competitive Adsorption of Multi-Metal

In order to evaluate the adsorption performance of UBC in multiple heavy metal complex pollution, the adsorption experiment of UBC on simulated wastewater with common metal ions (including Cu^2+^, Pb^2+^, Cd^2+^, Cr^3+^, and Fe^3+^) was carried out (Figure S3). The adsorption capacities of UBC for the five heavy metal ions were higher than that of BC. The adsorption capacities for Cr^3+^ and Fe^3+^ by UBC were higher than that by BC, and the maximum adsorption capacities reached 497.8 mg/g and 499.3 mg/g, respectively, with removal rates close to 100%. The adsorption capacities of Cu^2+^ and Pb^2+^ were 424.6 mg/g and 419.1 mg/g with removal rates of 80%. The adsorption capacity of Cd^2+^ was only 70.3 mg/g. Furthermore, UBC had a stronger affinity for Cr^3+^, Fe^3+^, Cu^2+^, and Pb^2+^. It might be related to the electronegativity of pollutants themselves [16,32]. The above experimental results showed that UBC is an adsorbent that could efficiently adsorb a variety of heavy metal ions and has great potential for treating heavy metal ion wastewater.

### 3.5. Comparison with Adsorption Capacity of Other Biopolymer Adsorbents

**Figure 4:**
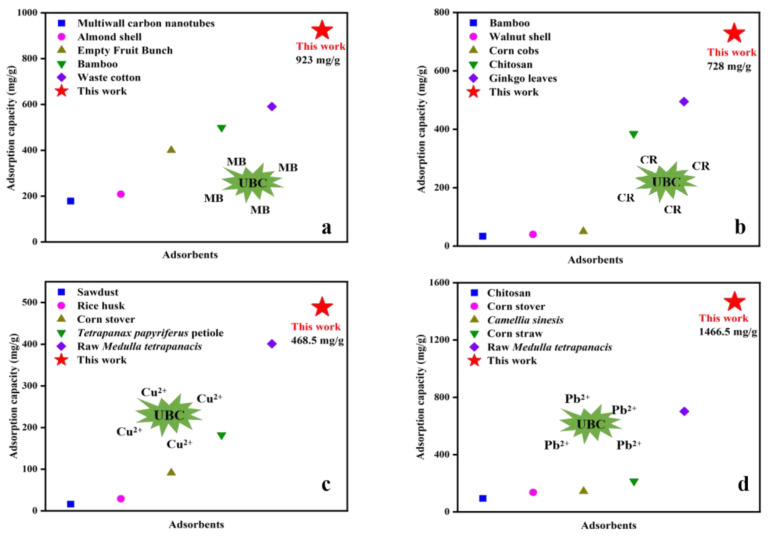
Adsorption capacity of various biomass adsorbents for MB (**a**), CR (**b**), Cu^2+^ (**c**), and Pb^2+^ (**d**).

Biomass adsorbents for removing heavy metal ions and dyes that have been prepared from various biomass materials are shown in Figure 4. Biomass adsorbents fabricated from multiwall carbon nanotubes [11], almond shell [31], empty fruit bunch [39], bamboo [29], and waste cotton [34] were used for the adsorption of MB; the maximum adsorption capacities were 178.5, 208.3, 400.0, 499.3, and 590.7 mg/g, respectively. The adsorption capacities of biochar for CR were illustrated, including bamboo (33.7 mg/g) [41], walnut shell (40.0 mg/g) [42], corn cobs (50.0 mg/g) [43], chitosan (384.6 mg/g) [44], and ginkgo leaves (495.0 mg/g) [45]. *Tetrapanax papyriferum* petiole biochar (182.0 mg/g) [8], rice husk (29.1 mg/g) [19], corn stover (91.2 mg/g) [20], sawdust (16.1 mg/g) [27], and raw *Medulla tetrapanacis* (430.9 mg/g) [10] were used to adsorb Cu^2+^ from aqueous solution. Biochar derived from corn stover [20], *camellia sinesis* [28], chitosan [28], corn straw [26], and raw *Medulla tetrapanacis* [10] were applied to adsorption Pb^2+^; the equilibrium adsorption capacities were 135.8, 143.9, 94.0, 214.0, and 701.6 mg/g, respectively. In the present study, adsorption capacities of UBC were higher than that of other biomass adsorbents, which were 923.0 (MB), 728.0 (CR), 468.5 (Cu^2+^), and 1466.5 (Pb^2+^) mg/g. Therefore, the UBC obtained by N-doped from *Medulla tetrapanacis* could be because the efficient adsorbent removes heavy metal ions and dyes in wastewater.

### 3.6. Regeneration Study

In this experiment, Cu^2+^ and Pb^2+^ were re-adsorbed for five cycles to test the regeneration ability of UBC. The adsorption capacity of UBC after five desorption regeneration cycles (Figure S4) showed that the removal efficiency of UBC for Cu^2+^ and Pb^2+^ was decreased as the number of cycles increased. In the second cycle, the adsorption capacity of Cu^2+^ and Pb^2+^ dramatically reduced, which decreased to 172.9 mg/g and 558.4 mg/g, respectively. After the third adsorption test, the adsorption efficiency of UBC was decreased slightly. After five cycles, UBC still has strong adsorption capacity for Cu^2+^ and Pb^2+^. The desorption regeneration test results showed that UBC had good reusability and practical value.

### 3.7. Adsorption Mechanisms

The UBC exhibited high adsorption capacity; the adsorption mechanism probably involved electrostatic attraction, pore physical adsorption, π-π conjugate interaction, complexation, hydrogen bonding, and ion exchange surface precipitation or co-precipitation [12,19,28].

The morphology and elementary composition of UBC-adsorbed dyes and heavy metal ions were revealed from SEM-EDS (Figure S5). The SEM images displayed that the biochar still maintained a complete framework and porous structure after adsorption, which laid the foundation for recycling of UBC. There were particles attached to carbon pores after UBC adsorption, indicating precipitation and pore physical adsorption existed in the adsorption process of UBC. Elemental composition of EDS proved the effectiveness of UBC for adsorption of dyes and heavy metal ions. The S element in EDS spectra after UBC adsorption was derived from dye molecules. It could be seen the characteristic peaks were kept consistent at 1583 cm^−1^ and 1210 cm^−1^ from the FTIR spectra of after UBC adsorption for dye (Figure S2b), indicating that the functional groups contained in UBC took little part in the dye adsorption stage. In addition, physical adsorption of organic dyes by UBC relied on its excellent pore structure. When UBC adsorbed Cu^2+^ and Pb^2+^, the intensity of the absorption peaks at 1583 cm^−1^ and 1210 cm^−1^ was decreased [13,26]. It showed that the functional groups contained in UBC participated in the reaction during the adsorption process, and chemical adsorption was the chief adsorption mechanism of removing heavy metal ions by UBC.

**Figure 5:**
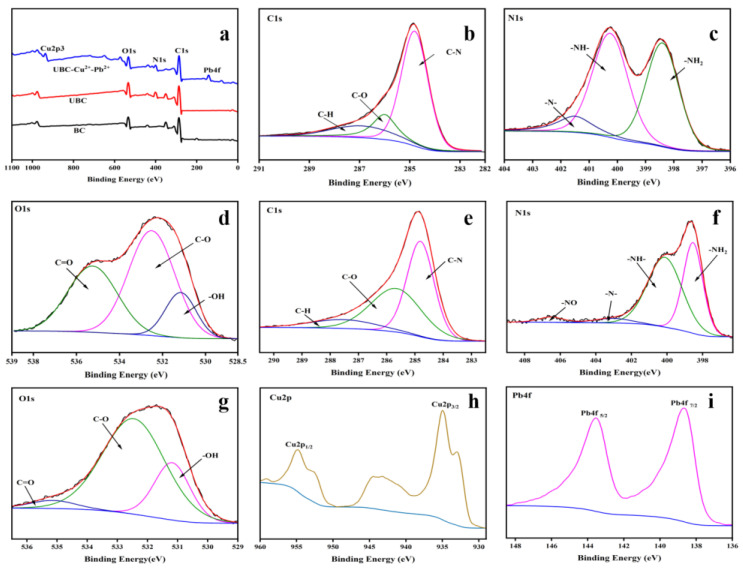
The XPS spectra of BC and UBC (**a**); the high-resolution XPS spectra of C1s, N1s and O1s of UBC (**b**–**d**); the XPS spectra of C1s, N1s, O1s, Cu2p, and Pb4f of UBC adsorbed for Cu^2+^ and Pb^2+^ (**e**–**i**).

The adsorption mechanism of UBC was further analyzed by comparing the XPS spectra of UBC before and after adsorption for Cu^2+^ and Pb^2+^. The peaks of C1s, O1s, and N1s of BC and UBC surface were detected by XPS scanning spectra (Figure 5a). Compared with BC, a strong new peak at 400 eV (N1s) could be observed in the spectrum of the UBC. The N1s spectra at 398.4 eV, 400.25 eV, and 401.5 eV were allotted to pyridonic nitrogen, pyrrolic N, and graphitic N, respectively [23,24]. There were C−H (287.01 eV), C−O (286.05 eV), C−N (284.81 eV), O−H (531.15eV), C−O (532.5 eV), and C=O (535.25 eV) in the C1s and O1s spectra of UBC, indicating that UBC contained a great quantity of nitrogen-containing and oxygen-containing functional groups [16,23]. As shown in Figure 5b–g, the peaks of primary amine and secondary amine were shifted slightly after the Cu^2+^ and Pb^2+^ were adsorbed by UBC, while the peak of tertiary amine shifted evidently from 401.5 eV to 403.1 eV in the fitting figure of N1s. It indicated that tertiary amine groups had a significant function in the chemical adsorption for heavy metal ions of UBC. Chemical adsorption is one of the adsorption mechanisms. A new −NO-fitting peak appeared at 406.55 eV, and the C=O peak in the O1s fitting peak decreased significantly, indicating that the N-containing group and carboxyl group participated in the adsorption. The disappearance of the Ca2p3 peak at 376 eV and the peak of Cu2p3 and Pb4f appeared at 944 eV and 144 eV in the XPS broad spectrum (Figure 5a), which suggested that Ca^2+^ sited in UBC might be replaced by substitution reaction during the adsorption process. The Cu2p and Pb4f peaks were convoluted to major bands at 144, 139, 955, and 935eV, corresponding to Cu2p_1/2_, Cu2p_3/2_ (Figure 5h), Pb4f_5/2,_ and Pb4f_7/2_ (Figure 5i), respectively [29,34,46]. It confirmed that Cu^2+^ and Pb^2+^ were adsorbed to UBC, indicating the presence of chemisorption in the adsorption process. In summary, ion exchange, precipitation, complexation, π-π interaction, and physical adsorption were involved in adsorption dyes and heavy metal ions on UBC.

## 4. Conclusions

Lignocellulosic biochar UBC were successfully prepared from *Medulla tetrapanacis* by pyrolysis using urea as a nitrogen source. The surface area and adsorption capacity were increased after being doped with urea. The UBC revealed monomolecular layer adsorption, and the adsorption progress conforms to pseudo-second-order dynamics with chemical adsorption. UBC can be recycled efficiently after five times reuse without a significant decrease in the loading capacity. Thus, the adsorption performance of biochar was significantly improved by the N-doping modification, and lignocellulosic biochar would be a good adsorbent for both organic dyes and heavy metal ions, which has good application prospects.

## Figures and Tables

**Table 1:** Physicochemical characteristics of biochars.

	BC	UBC_1−1_	UBC_1−2_		BC	UBC_1−1_	UBC_1−2_
C (%)	61.58	69.07	68.94	S_BET_ (m^2^/g)	198.51	707.56	1116.94
O (%)	36.87	19.27	16.96	Average pore volume (cm^3^/g)	0.15	0.51	0.75
N (%)	0.07	9.24	11.54	Micropore volume (cm^3^/g)	0.06	0.25	0.37
H (%)	1.48	2.42	2.56	Mesopore volume (cm^3^/g)	0.08	0.26	0.38

**Table 2:** Adsorption kinetics and intraparticle diffusion model parameters of UBC for metal ions and dyes.

	MB	CR	Cu^2+^	Pb^2+^
Pseudo-first-order				
*Q_e_* (mg/g)	192.42	559.53	154.58	423.18
*K*_1_ (min^−1^)	0.02	0.01	0.02	0.03
R^2^	0.89	0.96	0.87	0.94
Pseudo-second-order				
*Q_e_* (mg/g)	769.23	666.67	476.19	1250.00
*K*_2_ (min^−1^)	4.57 × 10^−4^	3.94 × 10^5^	2.78 × 10^−4^	1.83 × 10^−4^
R^2^	0.99	0.99	0.99	0.99
Intraparticle diffusion				
*K_i__d_*_,1_ (mg·g^−1^·min^−^^1/2^)	293.32	48.68	182.11	299.35
C_1_	−104.45	15.06	−218.00	13.51
R_1_^2^	0.95	0.96	0.93	0.95
*K_i__d_*_,2_ (mg·g^−1^·min^−^^1/2^)	29.32	22.72	4.82	48.25
C_2_	515.40	104.40	371.03	794.61
R_2_^2^	0.94	0.92	0.91	0.96
*K_i__d_*_,3_ (mg·g^−1^·min^−^^1/2^)	0.66	5.55	2.60	2.15
C_3_	734.47	464.00	406.19	1218.70
R_3_^2^	0.93	0.92	0.91	0.91

**Table 3:** Adsorption isotherm parameters for dyes metal ions on the UBC.

	Langmuir			Freundlich		
	*Q_max_* (mg/g)	*K_L_* (L/mg)	R_L_^2^	*K_F_* (mg/g)	*n*	R_F_^2^
MB	909.09	0.30	0.99	490.12	8.9969	0.98
CR	714.29	0.82	0.99	553.86	10.5164	0.85
Cu^2+^	526.32	0.20	0.99	395.18	10.7887	0.60
Pb^2+^	1666.67	0.02	0.99	115.77	5.8173	0.90

**Table 4:** Thermodynamic parameters for dyes and metal ions adsorption of UBC.

Sample	T (K)	ln*K_c_*	Δ*G^θ^* (kJ/mol)	Δ*H^θ^* (kJ/mol)	Δ*S^θ^* (J/mol/K)
MB	303	2.76	−6.95	9.34	27.51
	313	3.19	−5.30		
	323	2.31	−6.21		
CR	303	2.29	−5.77	6.73	4.58
	313	1.32	−3.43		
	323	1.98	−5.31		
Cu^2+^	303	1.47	−3.69	9.90	33.97
	313	0.91	−2.36		
	323	0.90	−2.42		
Pb^2+^	303	3.26	−8.21	−1.26	54.38
	313	3.37	−8.78		
	323	3.56	−9.55		

## Data Availability

The data presented in this study are available on request from the corresponding author.

## Supplementary Materials

The following are available online at https://www.mdpi.com/article/10.3390/polym14183889/s1. Detailed description of adsorption experiments, Figure S1: Scanning electron microscopy images of BC (a), UBC_1-1_ (b), UBC_1-2_ (c), Figure S2: FTIR spectra of biochar (a) and after adsorption of UBC (b), Figure S3: The adsorption capacity of mixed heavy metals solution by BC and UBC, Figure S4: The adsorption cycle diagram of UBC, Figure S5: SEM-EDS images of UBC after adsorption for MB (a), CR (b), Cu^2+^ (c), and Pb^2+^ (d), Table S1: Adsorption kinetics, isotherm models, and thermodynamics model of UBC adsorption.
