# Supplementary material for: N-Doped Biochar from Lignocellulosic Biomass for Preparation of Adsorbent: Characterization, Kinetics and Application
Source: Polymers (Basel). 2022 Sep 17;14(18):3889. doi: 10.3390/polym14183889 (PMC9503327; doi:10.3390/polym14183889)
Supplement: Supplementary file 1 [file polymers-14-03889-s001.zip › polymers-1878838-supplementary.pdf]

N-Doped Biochar from Lignocellulosic Biomass for Preparation of Adsorbent: Characterization, Kinetics and Application

Jing Li †, Fanxun Lv †, Ran Yang, Liping Zhang, Wei Tao, Guotao Liu, Hui Gao * and Ying Guan *

School of Forestry and Landscape Architecture, Anhui Agricultural University, Hefei 230036, China

* Correspondence: huigaozh@163.com (H.G.); xiaomi1231@163.com (Y.G.)

† These authors contributed equally to this study.

Figure captions

Figure S1. Scanning electron microscopy images of BC (a), UBC_{1-1} (b), UBC_{1-1} (c).

Figure S2. FTIR spectra of biochar (a) and after adsorption of UBC (b).

Figure S3. The adsorption capacity of mixed heavy metals solution by BC and UBC.

Figure S4. The adsorption cycle diagram of UBC.

Figure S5. SEM-EDS images of UBC after adsorption for MB (a), CR (b), Cu^{2+} (c) and Pb^{2+} (d).

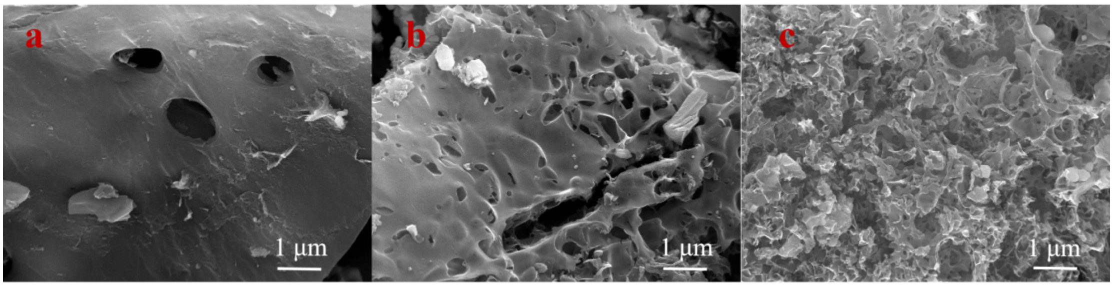

Figure S1. Scanning electron microscopy images of BC (a), UBC_{1-1} (b), UBC_{1-1} (c).

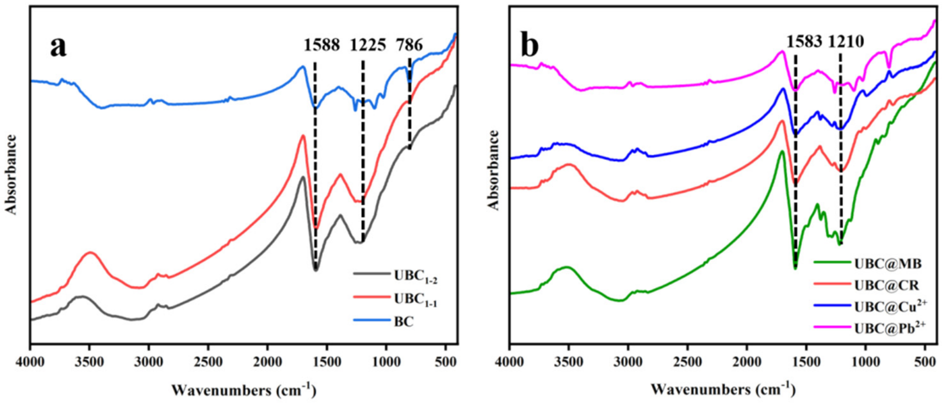

Figure S2. FTIR spectra of biochar (a) and after adsorption of UBC (b).

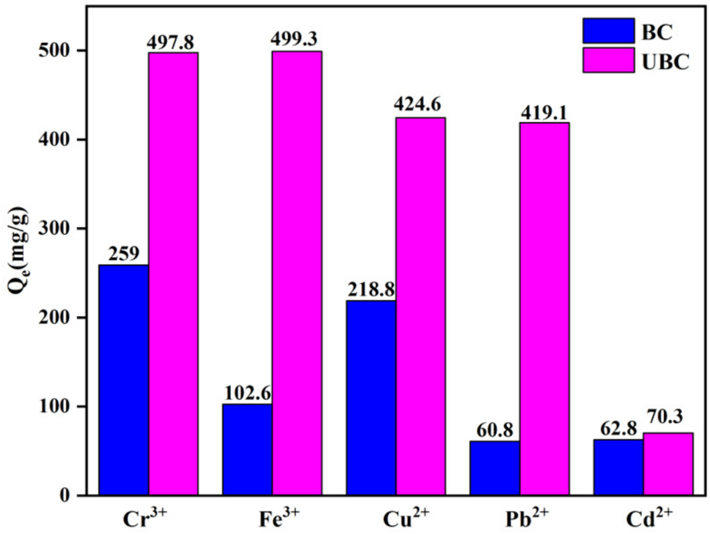

Figure S3. The adsorption capacity of mixed heavy metals solution by BC and UBC.

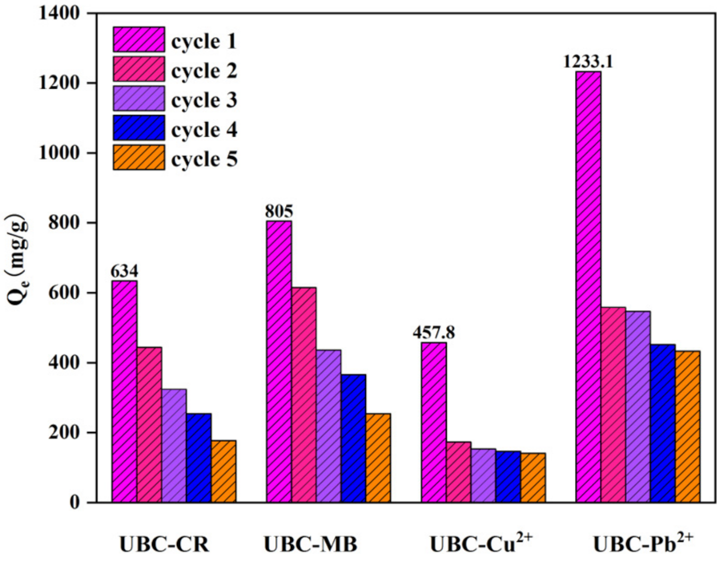

Figure S4. The adsorption cycle diagram of UBC.

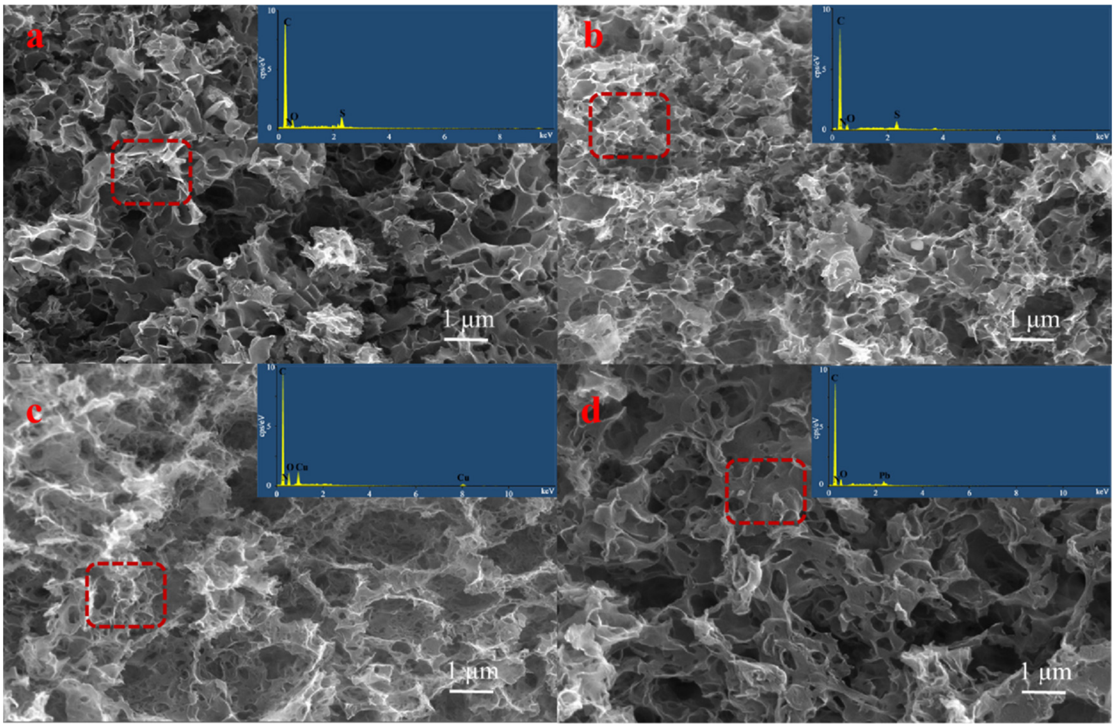

Figure S5. SEM-EDS images of UBC after adsorption for MB (a), CR (b), Cu^{2+} (c) and Pb^{2+} (d).

Table captions

Table S1. Adsorption kinetics, isotherm models and thermodynamics model of UBC adsorption.

Table S1. Adsorption kinetic, isotherm models and thermodynamics model of UBC adsorption.

Model	Equation	Definition
Kinetic models		
Pseudo-first-order	$\ln(Q_e - Q_t) = -K_1 t + \ln Q_e$	where Q_e and Q_t (mg/g) represent the adsorption capacity at adsorption equilibrium and time t (min); K_1 (min^{-1}), K_2 ($g \cdot mg^{-1} \cdot min^{-1}$), and K_{id} ($mg \cdot g^{-1} \cdot min^{-1/2}$) are the rate constants of the pseudo-first-order model, the pseudo-second-order model, and the intraparticle diffusion model, respectively.
Pseudo-second-order	$t/Q_t = 1/(K_2 Q_e^2) + t/Q_e$	
Intra-particle diffusion	$Q_t = K_{id} t^{1/2} + C$	
Isotherm models		
Langmuir	$C_e/Q_e = C_e/Q_{max} + 1/(K_L Q_{max})$	where C_e (mg/L) is the equilibrium concentration; Q_e and Q_{max} (mg/g) are the equilibrium adsorption and the maximum adsorption capacity; K_L (L/mg) is the Langmuir adsorption constant; K_F and $1/n$ are the Freundlich adsorption constants.
Freundlich	$\lg Q_e = \lg K_F + 1/n * \lg C_e$	
Thermodynamics model		
	$\Delta G^\theta = -RT \ln Kc$	Where R (8.314 J/K mol) is the gas constant; T (K) is adsorption temperature; Kc is the thermodynamic equilibrium constant; ΔG^θ (kJ/mol), ΔH^θ (kJ/mol) and ΔS^θ (J/mol/K)represent Gibbs free energy change, entropy change and enthalpy change, respectively.
	$\ln K_c = \Delta S^\theta/R - \Delta H^\theta/(RT)$	
	$K_c = Q_e/C_e$	
